# Phylogenetic Analysis of Entomoparasitic Nematodes, Potential Control Agents of Flea Populations in Natural Foci of Plague

**DOI:** 10.1155/2014/135218

**Published:** 2014-04-03

**Authors:** E. I. Koshel, V. V. Aleshin, G. A. Eroshenko, V. V. Kutyrev

**Affiliations:** ^1^Russian Research Anti-Plague Institute “Microbe”, Saratov 410005, Russia; ^2^Belozersky Institute of Physical-Chemical Biology, Lomonosov Moscow State University, Moscow 119991, Russia; ^3^Institute for Information Transmission Problems, Russian Academy of Sciences, Moscow 127994, Russia; ^4^National Research Institute of Physiology, Biochemistry, and Nutrition of Farm Animals, Russian Academy of Agricultural Sciences, Kaluga Region, Borovsk 249013, Russia

## Abstract

Entomoparasitic nematodes are natural control agents for many insect pests, including fleas that transmit *Yersinia pestis*, a causative agent of plague, in the natural foci of this extremely dangerous zoonosis. We examined the flea samples from the Volga-Ural natural focus of plague for their infestation with nematodes. Among the six flea species feeding on different rodent hosts (*Citellus pygmaeus*, *Microtus socialis*, and *Allactaga major*), the rate of infestation varied from 0 to 21%. The propagation rate of parasitic nematodes in the haemocoel of infected fleas was very high; in some cases, we observed up to 1,000 juveniles per flea specimen. Our study of morphology, life cycle, and rDNA sequences of these parasites revealed that they belong to three distinct species differing in the host specificity. On SSU and LSU rRNA phylogenies, these species representing three genera (*Rubzovinema*, *Psyllotylenchus*, and *Spilotylenchus*), constitute a monophyletic group close to Allantonema and Parasitylenchus, the type genera of the families Allantonematidae and Parasitylenchidae (Nematoda: Tylenchida). We discuss the SSU-ITS1-5.8S-LSU rDNA phylogeny of the Tylenchida with a special emphasis on the suborder Hexatylina.

## 1. Introduction

More than 150 species of fleas feeding on different mammalian hosts, primarily rodents, are vectors of the bacterium* Yersinia pestis*, a causative agent of plague [1, 2]. In natural foci of plague, the dynamics of flea populations are among the main factors controlling the incidence of epizootics that pose a threat to humans inhabiting the areas [3–5]. Entomoparasitic nematodes of the order Tylenchida are known to control populations of various insect hosts [6–9]. The rate of tylenchid infestation in fleas reaches 50–60% in some cases [10, 11], when the nematodes cause castration and early death of the flea hosts [9, 12, 13].

Despite high importance of the Tylenchida as a nematode order harboring entomoparasites and notorious crop pests, their reliable phylogeny is still a challenge. Tylenchid nematodes differ widely in life cycle, parasitic strategies, and the host range that spans plants, fungi, and invertebrates. Phylogenies obtained from SSU and partial LSU rDNA data often disagree with classifications based on morphology and life cycle [14–21]. Phylogenetic resolution inside the order is far from being clear, which in many respects results from the insufficiency of data available to adequately describe its diversity. As for tylenchid parasites of fleas, only 31 species are described to date [9, 22–31], with no molecular vouchering. Here we present a study of parasitic nematodes isolated from fleas sampled from different rodent hosts in a natural focus of plague.

## 2. Materials and Methods

### 2.1. Collection of Samples

**Figure 1:**
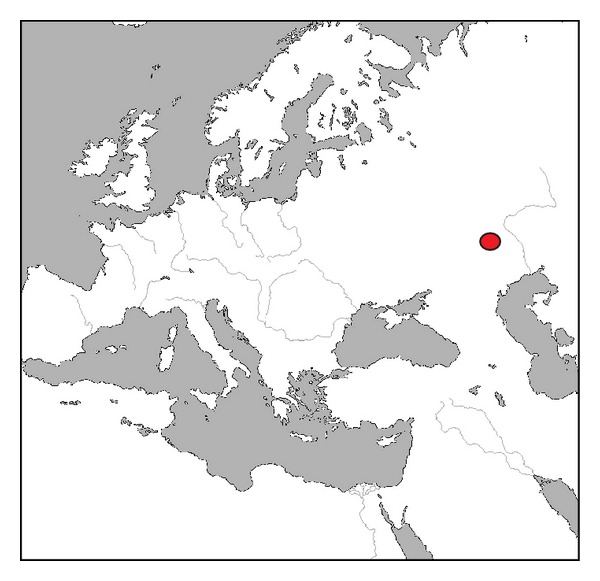
The sampling region on the map of Europe.

Samples were collected in 2012 (spring and autumn) and 2013 (spring) in the Volga-Ural natural focus of plague (Figure 1). The sampled rodents included sousliks (*Citellus pygmaeus*), mouse-like rodents (*Microtus socialis* and* Apodemus uralensis*), and jerboas (*Allactaga major*). Three flea species (*Citellophilus tesquorum*,* Neopsylla setosa*, and* Frontopsylla semura*) were sampled on sousliks; two species (*Amphipsylla rossica* and* Ctenophthalmus secundus*) were on* M. socialis* voles; and one species (*Mesopsylla hebes*) was on jerboas. Fleas were examined for nematode infestation (Table 1). Examination and dissection of fleas were carried out using the dissecting microscope MBS-2 (LOMO, Russia). A half of parasitic nematodes sampled from each flea was preserved for subsequent DNA extraction, and another half was used for morphological analysis. Live fleas infected with nematodes were placed in glass flasks with river sand to obtain free-living forms. Insects were kept in a KBF 720 (E5.2) climate chamber (Binder, Germany) at 26°C and 80% humidity.

### 2.2. Morphological Analysis

Fixation and clarification of nematode preparations were performed using standard techniques described by De Grisse [32]. Material was mounted on slides in a drop of glycerin, bound by a paraffin circlet (http://pest.cabweb.org). Color staining of preparations was not performed. Morphometric analysis was conducted using the light microscope “Leica DM 1000” (Leica, Germany) with an eyepiece micrometer. Pictures of nematodes were taken with the microscope “DFC 425” (Leica, Germany). Published data on morphometrics [23, 25, 26] were used for comparison.

### 2.3. DNA Extraction, PCR, and Sequencing

DNA samples were extracted with a Diatom DNA Prep (IsoGen Lab, Russia). rDNA fragments were amplified using an Encyclo PCR kit (Evrogen, Russia) and primers given in Table 2. The amplified rDNA fragments were sequenced using an Applied Biosystems 3500xL DNA analyzer. Sequence reads were assembled with the CAP contig assembly program [33] and proofread with the BioEdit software [34]. For three isolates, almost complete sequences of 18S and 28S rRNA and complete sequences of 5.8 rRNA, internal transcribed spacers ITS1 and ITS2 were assembled. The sequences were submitted to GenBank under accession nos. KF155281–KF155283. For the rest of isolates, partial (750–800 bp) sequences of 18S and 28S rRNA genes were submitted to GenBank under accession nos. KF373731–KF373740.

### 2.4. Phylogenetic Analysis

The newly obtained rDNA sequences of tylenchid parasites of fleas were aligned with a selected set of other tylenchid sequences obtained from the GenBank. The main selection criterion was to sample representatives of all clades that occur in published SSU and LSU rDNA phylogenies of the Tylenchida [16–21, 39]. Apart from the D2-D3 LSU rDNA expansion segment commonly used in previous studies, we included all LSU rDNA sequence data available for the Tylenchida, with the exception of* Basiria* sp. SAN-2005 (accession nos. DQ145619, DQ145667) that in our preliminary analyses (data not shown) demonstrated a disputable affinity to the Tylenchida. For the species* Anguina tritici*,* Globodera pallida*,* Heterodera glycines*,* Pratylenchus vulnus*, and* Radopholus similes* the nearly complete rDNA sequences were assembled with appropriate cDNA fragments identified with BLAST [40]. Partial LSU rDNA sequence of* Ditylenchus dipsaci* was combined with the soil environmental clone NTS_28S_061A_2_b4 (accession no. KC558346), as the clone sequence appeared to represent a close tylenchid relative of* D. dipsaci.* Chimeric sequences were also created in some cases when closely related partial rDNA sequences were found in the database. All sequences and their accession numbers are listed in Table 3. Cephalobidae and Chambersiellidae were chosen as the outgroup. Alignments were constructed with the MUSCLE program [41] and refined manually using the MEGA 5.0 software package [42]. Three alignments were generated: (1) SSU rDNA, (2) D3 region of LSU rDNA, and (3) concatenated rDNA data including SSU, LSU, 5.8S rDNA, and highly conserved regions of ITS1. After discarding ambiguously aligned positions, the alignments length was 1,723, 592, and 4,930 positions, respectively. Bayesian reconstruction of phylogeny was done with the PhyloBayes software, version 3.2 [43] under the GTR + CAT + DP model [44]. Eight independent runs were performed with 4,000,000 cycles each; the first 3,000,000 cycles were discarded. A consensus tree with Bayesian posterior probabilities was constructed for the remained tree sample. Bayesian reconstruction was also performed using the MrBayes software [45] under the GTR + G8 + I model [46] in two independent runs, each with four Markov chains. The chains were run for 5,000,000 generations, with trees sampling every 1,000th generation. The consensus posterior probabilities were calculated after discarding the first 3,000,000 generations. Partitioning “by genes” was used for the concatenated alignment with all parameters unlinked, except for the topology and branch lengths. In addition, node support was estimated with maximum likelihood bootstrap as implemented in the RAxML software, version 7.2.6 [47], under the GTR + G + I model with 1,000 bootstrap replicates. Alternative topologies were tested using the approximately unbiased (AU) [48] and Kishino and Hasegawa [49] tests implemented in the CONSEL software [50] and the expected likelihood weight test [51] implemented in the TREE-PUZZLE software [52]. TREEVIEW [53] was used as the tree viewer and editor, and site-wise log-likelihoods were computed with TREE-PUZZLE under the GTR + G8 + I model with substitution matrix parameters estimated by MrBayes.

## 3. Results

### 3.1. Infestation of Fleas with Nematodes

The infestation rate is shown in Table 1 (in total, 807 flea specimens were studied). Among the six flea species studied, the population size and the percentage of infected fleas varied depending on the season. Three flea species sampled on sousliks (*Citellophilus tesquorum*,* Neopsylla setosa,* and* Frontopsylla semura*) exhibited a stable population density. In the two species,* N. setosa* and* F. semura*, the infestation rate was moderate to high in the spring seasons of 2012 and 2013. In* C. tesquorum*, no infected fleas were detected in spring 2013, whereas in spring 2012 the fleas were highly infested (17.1%). The vole flea* Amphipsylla rossica* was abundant and moderately infested in autumn, whereas being less abundant in spring, which may explain the absence of infected fleas in the spring sample. Another vole flea,* Ctenophthalmus secundus*, exhibited a consistently high population density and low infestation rate in both spring and autumn samples.

**Figure 2:**
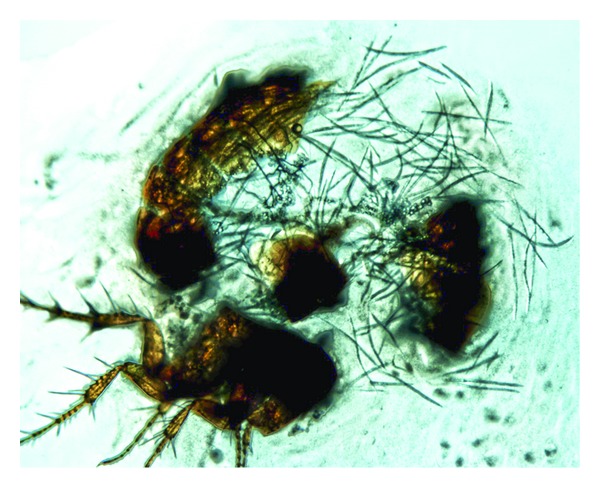
Numerous juveniles of* Rubzovinema* sp. extracted from the dissected body of a* Citellophilus tesquorum* flea.

Adult parasitic females and their progeny were found in the haemocoel of infected fleas. In the infected fleas* C. tesquorum*,* A. rossica*,* C. secundus*, and* Mesopsylla hebes*, only one generation of parasitic females was observed. Their amount in a flea specimen is determined by the number of free-living infective females that penetrate into the flea larva. We observed 1 to 2 or 1 to 4 adult parasitic females per flea specimen in spring and autumn, respectively. An additional parthenogenetic generation of parasitic females was found in some fleas of* N. setosa* and* F. semura*, where up to 16 specimens per flea were observed. As in other entomoparasitic nematodes, the propagation rate depends on the host age. Thus, in young fleas up to 10 juveniles was found per flea specimen, whereas up to 1,000 juveniles of different stages were contained in some old fleas (Figure 2). After the 2nd molt the number of juveniles is maximal, and 3rd stage juveniles massively migrate to the rectal section of the flea intestine for exit to the environment. In some cases, the observed infestation level was so high that nematodes penetrated distal segments of the flea legs, from where they have no way to the environment.

### 3.2. Morphological Analysis of Entomoparasitic Stages in Nematode Isolates and Their Taxonomic Identification

**Figure 3:**
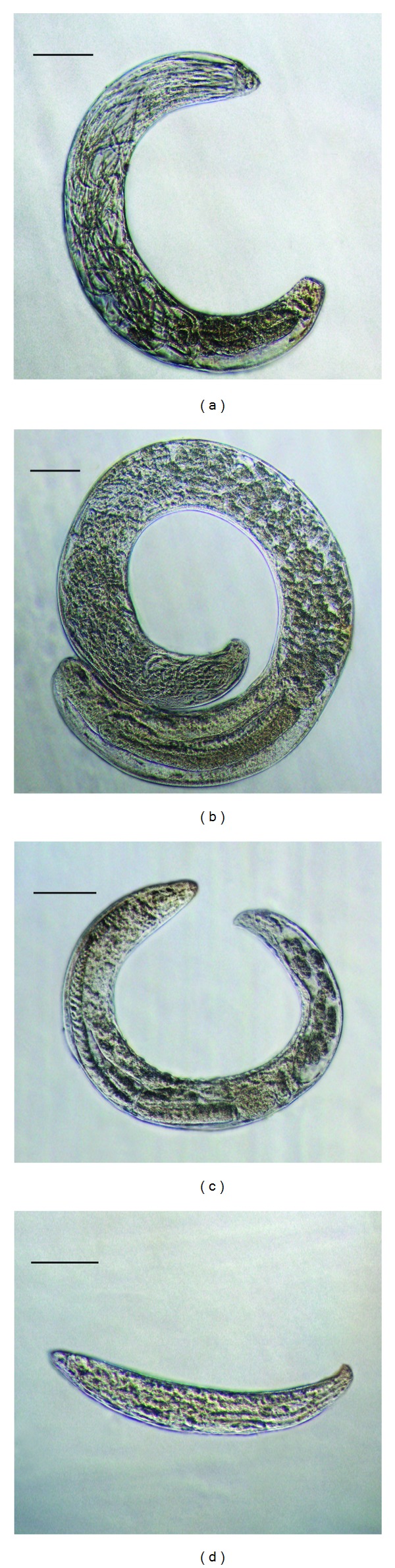
Parasitic females of the studied nematode species. (a)* Rubzovinema* sp., heterogeneous female; (b)* Spilotylenchus* sp., heterogeneous female; (c) *Psillotylenchus* sp., heterogeneous female of the first generation; (d) (c):* Psillotylenchus* sp., parthenogenetic female of the second generation. Scale bar—200 *μ*m.

**Figure 4:**
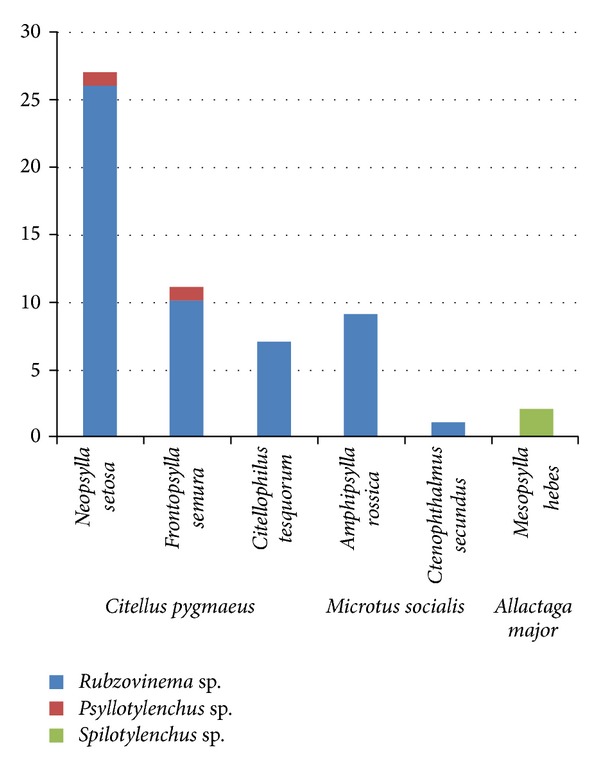
Distribution of the studied nematode species among the flea species studied, whose rodent hosts are given below. The vertical axis shows the numbers of infected fleas.

Analysis of morphology of entomoparasitic stages suggests that the studied nematode isolates from three distinct groups. A single generation of parasitic females was observed in the first two groups and an additional parthenogenetic generation—in the third group. According to morphometric data on adult parasitic females (Tables 4–6), the first two groups belong to the genera* Rubzovinema* or* Spilotylenchus* and the third group to the genus* Psyllotylenchus*. Photographs of parasitic females of* Rubzovinema* sp.,* Spilotylenchus* sp., and* Psyllotylenchus* sp. are depicted in Figure 3.  Figure 4 shows their distribution among flea samples studied.

According to morphometric evidence, parasitic females and juveniles of the genera* Rubzovinema* and* Spilotylenchus* are very similar. However, in the first two groups of isolates we found characters bearing discriminative and identificational value. In particular, the oesophageal glands in juveniles III of the first group are poorly developed. This is a distinctive feature of the genus* Rubzovinema*, where males and females have shortened oesophageal glands located close to the nerve ring. In the second group of isolates, oesophageal glands are well developed and elongated, which is characteristic of the genus* Spilotylenchus*. In the first group, the stylet possesses a heavily sclerotized distal spear with a length of approximately half the total stylet length and has a stem with a weaker sclerotization and widening to the base. This stylet structure is characteristic of the genus* Rubzovinema*, and stylet length (18.5 (14–22) *μ*m) is in accordance with morphometrics given in the description of this genus [26]. In the genus* Spilotylenchus,* the stylet varies in shape but always possesses a shortened conical distal spear. In the second group of isolates, the stylet structure was similar to that of* Spilotylenchus*. Also, the vulval lips of the first group are more protruded than in* Spilotylenchus*. Other features, including the morphometrics, vary widely in both genera, which hampers taxonomic identification. Nevertheless, based on distinctive traits, we identified the first and second group of isolates as* Rubzovinema* sp. and* Spilotylenchus* sp., respectively.

In the genus* Rubzovinema*, the single species described to date is* Rubzovinema ceratophylla* [26]. This species is known to parasitize exclusively the flea* Citellophilus tesquorum* that feeds on sousliks. The specimens of* Rubzovinema* studied in this work were isolated from five flea species,* C. tesquorum*,* Neopsylla setosa*,* Frontopsylla semura*,* Amphipsylla rossica*, and* Ctenophthalmus secundus*, of which the latter two were sampled on mouse-like rodents. Also, the parasitic females of* Rubzovinema* sp. differed from* R. ceratophylla* by morphology; they have a shorter tail and more protruded vulval lips. A morphometric comparison of* Rubzovinema* sp. and* R. ceratophylla* is given in Table 4.

The parasitic females of* Spilotylenchus* sp. were isolated from the flea* Mesopsylla hebes* associated with jerboas. The females were not identified to the species level because of a small number of available specimens and the lack of a free-living stage. A morphometric comparison of* Spilotylenchus* sp. and the morphologically closest species* Spilotylenchus maisonabei* [23] is given in Table 5.

In the genus* Psyllotylenchus*, descriptions of most species are fragmentary and incomplete, which precluded the species identification of the* Psyllotylenchus* isolates from the fleas* N. setosa *and* F. semura* feeding on sousliks. A morphometric comparison of* Psyllotylenchus* sp. and the type species of this genus,* Psyllotylenchus viviparous* [25], is given in Table 6.

The 18S and 28S rDNA sequences of* Rubzovinema* sp. specimens from* A. rossica* and* C. secundus *were 100% identical, which indicates that the isolates belong to the same species. The sequences of* Rubzovinema* sp. ex* C. tesquorum*,* Rubzovinema* sp. ex* N. setosa*, and* Rubzovinema* sp. ex* F. semura *diverged from one another and from the gene sequences of* Rubzovinema* sp. ex* A. rossica *and* Rubzovinema* sp. ex* C. secundus* by 0.4–0.7%, which corresponds to the levels of intraspecific variation [14, 114–119]. The 18S and 28S rDNA sequences of* Psyllotylenchus* sp. ex* N. setosa* and* Psyllotylenchus* sp. ex* F. semura* were 100% identical, indicating that they belong to the same species. The 18S and 28S rDNA sequences of* Rubzovinema* sp. and* Psyllotylenchus* sp. diverge by 1.2% and 1.9%, respectively. Those of* Spilotylenchus* sp. ex* M. hebes* were found to be more divergent. The degree of divergence of the 18S rDNA sequence of* Spilotylenchus* sp. ex* M. hebes* from those of either* Rubzovinema* sp. or* Psyllotylenchus* sp. was 2.4%; the D3 expansion segment of 28S rDNA diverged by 13.1% and 12.0%, respectively. The observed divergence rate of rDNA sequences agrees well with published evidence on entomoparasitic nematodes [14, 114–118]. Thus, intraspecific divergence of 18S rDNA in* Deladenus siricidicola* is 1% [120], of D2 and D3 expansion segments in the phytoparasite* Bursaphelenchus xylophilus* is from 0% to 0.6%, and the interspecific variation between the phytoparasites* B. xylophilus* and* Bursaphelenchus mucronatus* is from 1.7% to 3.7%. The spacers ITS1 and ITS2 are generally more diverged; the intra- and interspecific variation for these species is from 0 to 3.1% and 11.2 to 13.4%, respectively [121–123].

Molecular vouchering is proved to efficiently complement morphological species identification in nematodes [73, 122, 124–128]. Combining the rDNA and morphological data confirms the species identity within each of the three studied groups of isolates.

### 3.3. Phylogenetic Analysis

**Figure 5:**
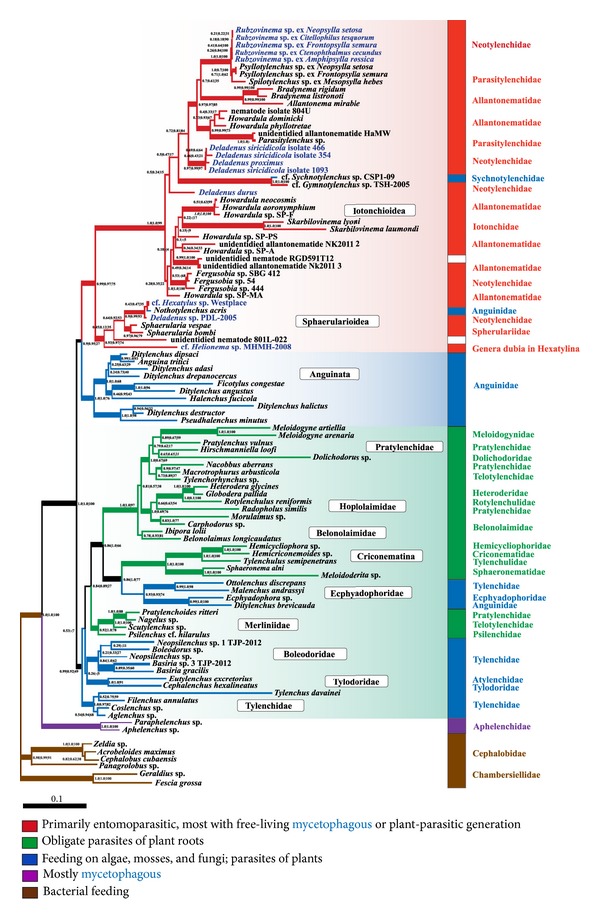
Phylogenetic tree of Tylenchida, inferred from SSU-ITS1-5.8S-LSU rDNA sequences. Topology was inferred using the PhyloBayes software (maxdiff = 0.36). Node support values are shown as follows: the first two values are Bayesian posterior probability assessed using the PhyloBayes and MrBayes software, respectively, and the third is bootstrap support assessed by the ML method. Thick lines lead to the nodes, in which at least one support value of posterior probability is 0.95 and higher. Names of clades (framed) are mainly given by type genera included in them (with the exception of Iotonchioidea). Formal taxonomic position (family by [8]) is shown on the right to the color bar. Colors indicate the ecologies (see the legend). Names of the species of Hexatylina that have a mycetophagous stage in their life cycle are shown in blue. The three robust major branches of Tylenchida are marked by gradient.

In phylogenetic analyses of rDNA we used a dataset with extensive species and gene sampling (SSU-ITS1-5.8S-LSU) compared to earlier published tylenchid phylogenies, most of which were based on SSU rDNA or D2-D3 expansion segments [17, 19–21, 39, 129]. The SSU-ITS1-5.8S-LSU rDNA tree topology (Figure 5) is highly similar to other published phylogenies of tylenchids. In this tree, tylenchomorphs are represented by the sister groups Aphelenchidae and Tylenchida. Most of the tylenchid clades occur in published trees but often contradict classifications based on morphology, as it was also noted by other authors [17, 19–21, 39, 129]. The three robust major branches in the SSU-ITS1-5.8S-LSU rDNA tree (Bayesian posterior probabilities of 0.99–1.0) are (1) the clade includes representatives of the suborders Hoplolaimina, Criconematina, and Tylenchina (excluding Anguinoidea); (2) the majority of classic Anguinata; (3) the suborder Hexatylina. The studied parasites of fleas form a monophyletic group (bootstrap support of 100%) within the Hexatylina.

**Figure 6:**
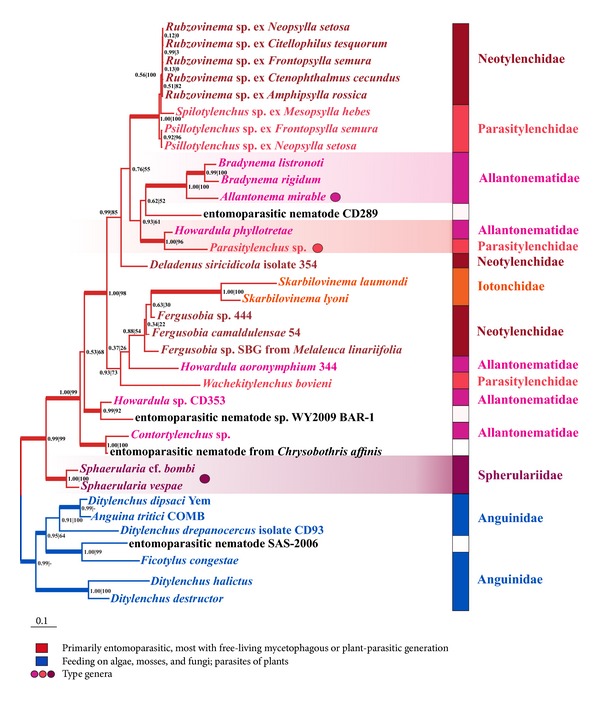
Phylogenetic tree of Hexatylina, inferred from D3 expansion segment of LSU rDNA. Topology was inferred using the PhyloBayes software. Node support values are shown as follows: Bayesian posterior probability/bootstrap support assessed by the ML method. Thick lines indicate the nodes supported at the level of 0.95 and higher. Color of lines indicates the ecologies (see the legend). Names of species were shown in different colors indicating their taxonomic position. Three families that include their type genera (shown as circles) are marked by gradient.

The nonredundant rDNA data on the Hexatylina in GenBank mostly represents the D2-D3 expansion segments of LSU rDNA. To maximize species sampling of the Hexatylina, we chose the D3 expansion segment as the molecular marker. The phylogenetic tree with the Anguinoidea as an outgroup is shown in Figure 6. In this tree, the suborder Hexatylina consists of two well-supported clades, in accordance with previously published D2-D3 rDNA phylogenies [19, 20, 39]. The clade of the studied flea parasites is placed within the largest branch of the Hexatylina, similarly to the result of the concatenated rDNA analysis.

The three alternative relationships between the three major branches of Tylenchida (Figure 5) are not discriminated by the AU and Kishino and Hasegawa tests, and only the basal position of the Hexatylina is rejected by the expected-likelihood weights test (Table 7). All three tests do not discriminate between the alternative placement of the flea parasites as closest to the* Allantonema*,* Parasitylenchus*, or* Deladenus* branches; however, its positioning outside this grouping is not rejected only by a less conservative Shimodaira-Hasegawa test [50].

## 4. Discussion

### 4.1. Ribosomal DNA Phylogeny of the Tylenchida and Relationships within the Suborder Hexatylina

Phylogenetic analyses of SSU [16, 17, 19, 39] and D2-D3 [20, 39] rDNA data using various methods and species sampling generally agree on the monophyly of most tylenchid clades and contradict classic morphology based classifications. In the SSU-ITS1-5.8S-LSU tree (Figure 5), the monophyletic Tylenchida consists of three major robust clades. The first clade diverges into six groups: (1) the “Tylenchidae (part 2)” (by [17]), (2) the Tylodoridae (represented by the two genera,* Cephalenchus* and* Eutylenchus* [83]), (3) Boleodorinae + “Tylenchidae (part 1)” (by [Bert]), (4) the Merliniidae [130], (5) Criconematina + Sphaeronematidae + selected Tylenchina, and (6) Belonolaimidae + “Hoplolaimina.” The Merliniidae group corresponds to Clade C in [19] and includes partially the polyphyletic “Telotylenchinae” [131], “Pratylenchidae”, and “Hoplolaimina” (*Psilenchus* cf.* hilarulus*). Group (5) corresponds to Clade 12A in [129], where Sphaeronematidae (*Sphaeronema* and* Meloidoderita*) were earlier shown to be closely related to Criconematina [20, 89], and selected Ecphyadophoridae +* Ottolenchus* +* Malenchus* were found to represent a monophyletic clade within the paraphyletic Tylenchina likely to be related to the Criconematina [18, 82]. Group (6) corresponds to Clade VII in [20], Clade 12B in [129], and Clade A + Clade B in [19]. Belonolaimidae (the genera* Belonolaimus* and* Ibipora*) tend to occupy the basal position. Clade A in [19] contains a “long branch” of the burrowing nematode* Radopholus similes* (“Pratylenchidae”) in sister position to the Hoplolaimidae [17, 19]. This nematode occupies a similar position relative to the Hoplolaimidae in the SSU-ITS1-5.8S-LSU tree, and we consider this unlikely to be an LBA artefact. Similarly to [95],* Carphodorus* and* Morulaimus* that belong to the classic Belonolaimidae comprise the basal branch of Clade A* sensu* [19]. The clade corresponding to Clade B in [19] contains Meloidogynidae, Dolichodoridae, paraphyletic Pratylenchidae, and a part of Telotylenchidae.

The second major clade of the Tylenchida includes representatives of the classic infraorder Anguinata, with a well-supported monophyletic origin, except for a few species. They belong outside the second clade and may initially have been wrongly identified.

The third major clade includes representatives of the classic suborder Hexatylina and consists of two groups. The smaller one unites the three species of* Sphaerularia*,* Helionema* sp., cf.* Hexatylus* sp.,* Deladenus* sp. PDL-2005, and* Nothotylenchus acris* (Anguinata: Nothotylenchidae). It is further referred to as the Sphaerularioidea according to the type genus. The larger group contains the clade of studied flea parasites and members of the superfamilies Iotonchioidea (*Skarbilovinema* spp.,* Parasitylenchus* spp., and* Wachekitylenchus bovieni*) and Sphaerularioidea (*Allantonema mirable*,* Bradynema* spp.,* Howardula* spp., and* Contortylenchus *sp. (fam. Allantonematidae);* Deladenus durus*,* Deladenus proximus*,* Deladenus siricidicola*,* Fergusobia* spp., and* Gymnotylenchus* sp. (fam. Neotylenchidae)). One species of the Anguinata,* Sychnotylenchus* sp., also joins the larger group. Our study renders the genera* Howardula* and* Deladenus* paraphyletic, as was earlier shown in [19, 39, 71, 119].

The genus* Howardula* is paraphyletic in published rDNA and mitochondrial COI phylogenies [71]. Such characters of* Howardula* as the degeneration of oesophagus, tail shape, and the absence of stylet in males seem to have evolved independently by convergence. The paraphyletic genus* Deladenus* is more closely related to either ancestral forms of the Hexatylina or forms typical to the Anguinata. The infraorder Anguinata includes soil-dwelling nematodes, mostly mycetophagous or parasitizing various parts of plants. However, an unidentified entomoparasitic nematode was also grouped within the Anguinoidea [39]. The life cycle of* Deladenus* spp. is an irregular alternation of free-living and entomoparasitic forms. The nematode* D. siricidicola* is able of producing an unlimited number of free-living generations in the absence of the host larvae of siricid pine-killing wood wasps [132]. Like in Anguinata, the free-living forms of* Deladenus* spp. are fungal feeding. Such characters of* Deladenus* asthe mycetophagy, enlargement of subventral glands in entomoparasitic females versus their reduction in free-living forms, the hypertrophy of dorsal glands, and stylet reduction in free-living forms seem to be symplesiomorphic. Resemblance with the Anguinata is also typical of other mycetophagous free-living forms:* Hexatylus* (Neotylenchidae),* Rubzovinema* (Neotylenchidae),* Prothallonema* (Sphaerularioidae)* Helionema* (Hexatylina* dubia*), and Paurodontidae. For the latter, the entomoparasitic stage is expected but has never been observed. The relationship between the Hexatylina and Anguinata was earlier hypothesized based on morphology [7, 8, 130, 133, 134]. On rDNA phylogenies of tylenchids, the monophyly of the Hexatylina + Anguinata is either supported [19] or not rejected [20]. In the SSU-ITS1-5.8S-LSUrDNA tree obtained in this study, the monophyly of the Hexatylina + Anguinata has the Bayesian posterior probability of 0.91, but the maximum-likelihood bootstrap support is low; the AU and Kishino and Hasegawa tests did not discriminate between alternative hypotheses.

According to our SSU-ITS1-5.8S-LSU rDNA phylogeny (Figure 5), the major robust branches of the Tylenchida are incongruent with morphology-based classifications suggesting three rather than four suborders (the rank is adopted from morphological systems of tylenchids). Among them, the Hexatylina and Anguinata (both are monophyletic) are likely to be sister groups. The third emerged suborder includes representatives of three classic suborders: Tylenchina, Hoplolaimina, and Criconematina, among which only the latter does not contradict morphology-based classifications.

Considering ecological traits coded in Figure 5, the mycetophagy and/or facultative ectophytoparasitism are likely to be ancestral in the Tylenchida. Sedentary phytoparasites (root-knot species of* Meloidogyne*, the false root-knot genus* Nacobbus*, and cyst-forming* Heterodera* and* Globodera*) and other obligate endoparasites of plants evolved several times from free-living or facultative sedentary forms, as it was previously hypothesized in accordance with the concept of evolutionary trend to endoparasitism in phytonematodes [135]. Similarly, obligate endoparasites of insects from the Hexatylina are likely to have evolved from mycetophagous forms, with some species retaining the ancestral mycetophageous stage in the life cycle (e.g., species of the paraphyletic genus* Deladenus* and flea nematodes of the genus* Rubzovinema*). An interesting specific case in the Hexatylina is the genus* Fergusobia* that includes plant parasites associated with insects [68, 70], which may have transited to plant parasitism via entomoparasitism [39].

### 4.2. Ribosomal DNA Phylogeny of the Flea Nematodes and Their Classification

The nematodes of fleas do not group with the families known as their relatives in morphology-based systems, as these families do not form monophyletic groups in the tree. However, they do group with both type genera of the families Parasitylenchidae and Allantonematidae (*Parasitylenchus* and* Allantonema*, resp.). This grouping is preceded by a successive divergence of* Deladenus durus* and* Deladenus siricidicola* (Figure 5). As mentioned above, the pronounced free-living form in* Deladenus* seems to be ancestral to this group.

Only 31 tylenchid species that parasitize in fleas have been described to date. They differ by morphology, life cycle, and the host specificity, and belong to the five genera:* Spilotylenchus* (8 species),* Psyllotylenchus* (20 species),* Incurvinema* (1 species)* Kurochkinitylenchus* (1 species), and* Rubzovinema* (1 species). According to the classification of Siddiqi [8], the genera* Spilotylenchus* and* Psyllotylenchus* belong to the family Parasitylenchidae, whereas the genus* Rubzovinema* is a member of the Neotylenchidae. The two families represent two superfamilies, Iotonchioidea and Sphaerularioidea, respectively. All rDNA phylogenies published to date suggest that these superfamilies are paraphyletic [19, 20, 39], which is also inferred in our study with an extensive gene and taxon sampling.

A high degree of rDNA similarity in the three studied species suggests a closer relationship of these species than that assumed by the accepted system of classification. Earlier, Slobodyanyuk proposed to unite all known flea parasites into one family, the Spilotylenchidae. Its four subfamilies, Spilotylenchinae, Rubzovinematinae, Psyllotylenchinae, and Kurochkinitylenchinae, are discriminated based on the life cycle features [28]. In Spilotylenchinae and Rubzovinematinae, the entomoparasitic stage is represented by parasitic females of one heterosexual generation. In Psyllotylenchinae, in addition to the heterosexual generation, a parthenogenetic generation occurs in the flea haemocoel. In Kurochkinitylenchinae, two heterosexual generations exist in the haemocoel: the first generation produces parasitic females and the second generation produces both females and males [28]. Siddiqi also considered the unification of all flea tylenchids into one family but observed the need for further evidence in support [8].

Our results strongly suggest the inclusion of the three genera,* Rubzovinema*,* Psyllotylenchus*, and* Spilotylenchus*, in one family, the Spilotylenchidae [28]. The ribosomal DNA genetic distance within the family Spilotylenchidae is much smaller than that of certain tylenchid genera, for example,* Meloidogyne* (Figure 4) or* Pratylenchus* [19, 84].

### 4.3. Host Specificity of Flea Nematodes

The majority of tylenchid nematodes are monoxenous or oligoxenous; in particular, flea parasites were thought to be strictly host specific. Earlier papers suggested the lack of strict host specificity in* Psyllotylenchus pawlowskyi* and* Psyllotylenchus viviparous* [13, 25]. However, later these species were found to be heterogeneous and sustained revision [9, 27–29].* Spilotylenchus pawlowskyi* and* Spilotylenchus caspius* were referred to as single-host parasites of the flea* Coptopsylla lamellifer* [27, 136].* Kurochkinitylenchus laevicepsi* and* Spilotylenchus ivashkini* also share the same flea host,* Nosopsyllus laeviceps* [28, 29]. Before our study, the genus* Rubzovinema* was known to contain a single species,* Rubzovinema ceratophylla*, which parasitizes exclusively the flea* Citellophilus tesquorum*.

We found that at least two out of the three studied species are not single-host parasites.* Psyllotylenchus* sp. was shown to parasitize two flea species feeding on sousliks,* Frontopsylla semura* and* Neopsylla setosa*.* Rubzovinema* sp. was found on five flea species feeding on different rodent hosts:* C. tesquorum*,* F. semura*,* N. setosa* (all sampled from sousliks),* Ctenophtalamus secundus*, and* Amphipsylla rossica* (all sampled from voles).* A. rossica, F. semura*, and* C. tesquorum *belong to different families of the superfamily Ceratophylloidea (Leptopsyllidae and Ceratophyllidae), whereas* C. secundus* and* N. setosa* belong to the superfamily Hystrichopsylloidea. Unlike the host-specific* R. ceratophylla*, the studied* Rubzovinema* sp. parasitizes taxonomically distant fleas feeding on different rodents. Thus, the common opinion that flea nematodes are strictly host specific should be revisited.

As the two species of* Rubzovinema* demonstrate, even closely related parasites may exhibit different host range size. Among other known examples are the entomoparasitic nematodes of the genus* Howardula* parasitizing various beetles and flies [71, 137, 138], many phytonematodes [8], sibling species of parasitoid flies [128], and herbivorous insects [139]. The host range of parasites is an indicator of their evolutionary strategy in the ecosystem. Multihost parasites can be considered ecological generalists, in contrast to specialists that coevolve with a particular host. Generalists and specialists play different roles in the ecosystem [140], where they keep in balance, taking advantages and disadvantages of the two strategies. The advantages of generalization are yet to be explained by evolutionary biologists, whereas advantages of specialization are obvious, and it is generally accepted that evolution favors specialism [141, 142]. In the flea parasites, this trend is demonstrated by a greater species diversity of ecological specialists, the genera* Spilotylenchus* and* Psyllotylenchus*.

Nevertheless, the generalist* Rubzovinema* sp. was most abundant in the studied samples, which indicates that extending the host range may be evolutionarily successful. Besides the need to combat the immune response of several hosts, which is a requirement to widen the hosts range [143], the free-living stage of* Rubzovinema* sp. is to adapt to diverse microbioclimatic conditions of complex environments of rodent habitats. Multihost parasites pay a cost of adapting to alternative conditions [141, 144] compensated by stable survival of the species. Considering the spatial and temporal dynamics of flea populations feeding on a particular rodent host (one or two flea species usually dominate over a sampling season), multihost nematode parasites gain an advantage of their relative independence of population waves of either flea hosts or their rodent hosts. A higher infestation rate observed for* Rubzovinema* sp., compared to the two other studied species, may be an indicator of a greater ecological plasticity of this multihost parasite.

### 4.4. Entomoparasitic Nematodes in Natural Foci of Plague

In natural foci of plague, the epizootic dynamics are influenced by numerous climatic and biotic factors. The spatial and temporal population dynamics of the plague agent,* Y. pestis*, affect the population dynamics of the flea vectors and their mammalian hosts. Members of the transmission route of the plague agent also closely interact with other living organisms. For example, parasites of fleas that in turn feed on rodents are hyperparasites that play the role of high-level control agents on the ecosystem level, the role that entomoparasitic nematodes share with the bacterial plague agent. High-level control agents render the epidemiological state of a natural focus of disease less predictable. On the one hand, a lower density of the flea vector population reduces the plaque transmission rate; on the other, its growth causes an exponential decay of the host rodent population [145] below its epidemiological threshold, above which there is a threat of spillover of plague infection into human population [145]. Hypothetically, nematode-induced decrease of flea population is able to increase the number of rodents above the threshold and thus trigger an epidemic. The dual effect of high-level control agents is well exemplified by cases, when during plague episodes the extermination of rodents by humans causes the return of infection through stimulating the migration of fleas, the plaque vectors [5].

The studied entomoparasitic nematodes possess high potential as control agents of the flea vectors of plague owing to their high propagation rate within the flea host (Figure 2) and high infestation level (up to 21% observed in this study and from 50 to 60%, as estimated by other authors [10, 11]). One of the studied nematode species,* Rubzovinema* sp., is a multihost parasite. Host-specific parasites reach the optimal level of pathogenicity by maintaining the trade-off between pathogenicity and transmissibility. Adding of a new host to a multihost system makes the model more complicated [141]. The multihost parasite* Rubzovinema* sp. is expected to exhibit different levels of pathogenicity with respect to different flea hosts which, in turn, play different roles in the transmission of plague. Epizootics cause sporadic mortality in local populations of all members involved in the interaction with the plague agent, and their survival is contingent on migrations within a metapopulation. It is the case when the Cope's law [139, 146] governs the extinction of specialists on a shorter time scale rather than a geological period, and evolution may favor the ecological generalists, such as* Rubzovinema* sp.

Some authors surmised the involvement of entomoparasitic nematodes in the transmission of the plague agent [4], as it was observed that biofilms of* Yersinia pestis* adhere to cuticle receptors of* Caenorhabditis elegans* [147–149]. In this perspective, nematodes parasitizing fleas in natural foci of plague take on greater importance, as they may provide for the transmission route that does not include a mammal [4]. Further studies will clarify the role of flea nematodes in the transmission of plague infection.

## Figures and Tables

**Table 1:** Number of fleas studied and the percentage of fleas infected with nematodes.

Time of sampling	Host rodent species	Flea species	Number of collected fleas	Number of infected fleas	Percentage of infected fleas
April 2012	*Citellus pygmaeus *	*Citellophilus tesquorum *	41	7	17.1%
		*Neopsylla setosa *	73	5	6.8%
		*Frontopsylla semura *	54	7	13%

October 2012	*Microtus socialis *	*Amphipsylla rossica *	135	9	6.7%
		*Ctenophthalmus secundus *	88	1	1.1%

April 2013	*Citellus pygmaeus *	*Citellophilus tesquorum *	34	0	0
		*Neopsylla setosa *	271	22	8.1%
		*Frontopsylla semura *	19	4	21%
	*Microtus socialis *and* Apodemus uralensis *	*Amphipsylla rossica *	6	0	0
		*Ctenophthalmus secundus *	52	0	0
	*Allactaga major *	*Mesopsylla hebes *	34	2	5.9%

**Table 2:** Nucleotide sequences of primers used in this study.

Primer	Sequence	Orientation	References
Nik22	tmycygrttgatyctgyc	F	This study
A	gtatctggttgatcctgccagt	F	[35]
Q5nemCh	gccgcgaayggctcattayaac	F	This study
G18SU	gcttgtctcaaagattaagcc	F	[36]
Ves18-d9	gtcgtaacaaggtatccgtaggtgaac	F	This study
R18Tyl1	ggtccaagaatttcacctctc	R	[36]
B	gtaggtgaacctgcagaaggatca	R	[35]
Q39nem	gaaaccttgttacgacttttrcbygg	R	This study
58d1	rcatcgatgaagaacgywg	F	[37]
58r nem	gcwgcgttcttcatcgacyc	R	This study
28d3	gtcttgaaacacggaccaagg	F	[37]
28d6	ggtyagtcgrtcctrag	F	[37]
D2A	acaagtaccgtgagggaaagttg	F	[38]
28r4	gctatcctgagggaaacttcgg	R	[37]
28r2nem	cggtacttgttcgctatcg	R	This study
28r7	agccaatccttwtcccgaagttac	R	[37]
28r12	ttctgacttagaggcgttcag	R	[37]
D3B	tcggaaggaaccagctacta	R	[38]

**Table 3:** List of OTUs and accession numbers of sequences.

Name	18S rRNA	ITS1-5.8S rRNA	28S rRNA	%, SSU-ITS1- 5.8S-LSU/D3	Reference	Family by [8]
**Chambersiellidae∗**						
*Fescia grossa *	KC242218	—	DQ145636 DQ145684	87.1/—	[54] [55]	Chambersiellidae
*Geraldius* sp. SAN-2010a	—	—	GU062821	17.8/—	[56]	Chambersiellidae

**Cephalobidae**						
*Acrobeloides maximus *	EU196016	JX026706	EU195987	94.8/—	[57] [58] [57]	Cephalobidae
*Cephalobus cubaensis *	AF202161	AF202161	EU253570	89.8/—	[59] [57]	
*Panagrolobus* sp. SN-2010	—	—	HM439771	51.9/—	[60]	
Cephalobidae Gen. sp. MHMH-2008	FJ040406	—	—		Holterman et al., 2008, unpublished.	
*Zeldia punctata *	—	DQ146426	EU195988	96.6/—	[61] [57]	
*Zeldia* sp.	AY284675	—	—			

**Aphelenchidae**						
*Aphelenchus avenae *	JQ348399	AF119048	—	96.9/—	[62] [63]	Aphelenchidae
*Aphelenchus* sp.	—	—	DQ145664 DQ145714		[55]	
*Paraphelenchus acontioides *	—	—	HQ218322	45.5/—	[64]	
*Paraphelenchus* sp.	AY284642	—	—		[18]	

**Hexatylina + “Anguinata (part)”: Iotonchioidae**						
*Allantonema mirable *	—	—	JX291132	10.6/85.8	[39]	Allantonematidae
*Bradynema listronoti *	DQ915805		DQ915804	45.6/96.8	[65]	
*Bradynema rigidum *			DQ328730	10.4/86.3	[20]	
*Contortylenchus* sp.	—	—	DQ328731	—/85.4	[20]	
*Deladenus durus *	JQ957898	—	—	34.0/—	[66]	Neotylenchidae
*Deladenus proximus *	JF304744	JF304744	—	35.2/—	[67]	
*Deladenus siricidicola* isolate 354	AY633447		AY633444	45.8/98.1	[68]	
*Deladenus siricidicola* isolate 466	FJ004890	FJ004890	—	41.7/—	[69]	
*Deladenus siricidicola* isolate 1093	FJ004889	FJ004889	—	42.0/—	[69]	
*Fergusobia camaldulensae *	AY589294	—	AY589346	45.7/98.0	[68]	
*Fergusobia* sp. 444	EF011667	—	EF011675	45.7/97.3	[68]	
*Fergusobia* sp. SBG	FJ393270	—	FJ386996	45.7/98.3	[70]	
cf. *Gymnotylenchus* sp. TSH-2005	AY912040	—	—	12.9/—	Powers et al., unpublished.	
*Howardula aoronymphium *	AY589304	AY589304	AY589395	49.7/96.1	[68]	Allantonematidae
*Howardula dominicki *	AF519234	AF519234	—	37.4/—	[71]	
*Howardula neocosmis *	AF519226	AF519226	—	38.2/—	[71]	
*Howardula phyllotretae *	JX291137	—	DQ328728	41.9/86.1	[39] [20]	
*Howardula* sp. CD353	—	—	JX291131	—/93.9	[39]	
*Howardula* sp. SP-A	AF519232	AF519232	—	37.7/—	[71]	
*Howardula* sp. SP-F	AF519222	AF519222	—	38.2/—	[71]	
*Howardula* sp. SP-MA	AF519233	AF519233	—	38.1/—	[71]	
*Howardula* sp. SP-PS	AF519231	AF519231	—	38.1/—	[71]	
*Parasitylenchus bifurcatus *	KC875397	—		44.0/85.3	[72]	
*Parasitylenchus* sp.	—	—	DQ328729		[20]	
*Psyllotylenchus* sp. ex *Frontopsylla semura *	KF373734	—	KF373739	27.1/93.7	This study	Parasitylenchidae
*Psyllotylenchus* sp. ex *Neopsylla setosa *	KF373733	—	KF373738	27.1/93.7	This study	
*Rubzovinema* sp.* ex Amphipsylla rossica *	KF155281	KF155281	KF155281	90.0/100.0	This study	Neotylenchidae
*Rubzovinema* sp. *ex Ctenophthalmus cecundus *	KF155282	KF155282	KF155282	89.8/100.0	This study	
*Rubzovinema* sp. *ex Citellophilus tesquorum *	KF155283	KF155283	KF155283	93.2/100.0	This study	
*Rubzovinema* sp. ex *Frontopsylla semura *	KF373732	—	KF373737	27.1/93.7	This study	
*Rubzovinema* sp. ex *Neopsylla setosa *	KF373731	—	KF373736	27.1/93.7	This study	
*Skarbilovinema laumondi *	—	—	JX291136	10.9/91.0	[39]	Iotonchioidea
*Skarbilovinema lyoni *	JX291138	—	DQ328733	41.8/86.3	[39] [20]	
*Spilotylenchus* sp. ex* Mesopsylla hebes *	KF373735	—	KF373740	27.1/93.4	This study	Parasitylenchidae
cf. *Sychnotylenchus* sp. CSP1-09	DQ080531	—	—	12.9/—	Powers et al., unpublished.	Sychnotylenchidae
*Wachekitylenchus bovieni *	—	—	DQ328732	—/85.9	[20]	Parasitylenchidae
Unidentified Allantonematidae HaMW	JQ941710	—	—	18.5/—	Rhule, unpublished.	Allantonematidae
Unidentified Allantonematidae NK2011_2	AB663183	—	—	12.0/—	[73]	
Unidentified Allantonematidae NK2011_3	AB663184	—	—	12.0/—	[73]	
Unidentified nematode 804U-025	EU880149	—	—	12.0/—	[74]	
Unidentified nematode CD289	—	—	JX291133	—/84.1	[39]	
Unidentified nematode RGD591T12	AB455970	—	—	12.0/—	[73]	
Unidentified nematode WY2009_BAR-1	—	—	FJ661075	—/96.3	[75]	
Unidentified parasite ex *Chrysobothris affinis *	—	—	DQ202658	—/51.0	Hunt et al., unpublished.	

**Hexatylina + “Anguinata (part)”: Sphaerularioidea**						
*Deladenus* sp. PDL-2005	AJ966481	—	—	35.0/—	[16]	Neotylenchidae
cf. *Helionema* sp. MHMH-2008	EU669913	—	—	34.0/—	[19]	Parasitylenchidae (genera dubia in Hexatylina)
cf. *Hexatylus* sp. Westplace	AY912050	—	—	12.9/—	Powers et al., unpublished.	Neotylenchidae
*Nothotylenchus acris *	AY593914	—	—	34.0/—	[76]	Anguinidae
*Sphaerularia bombi *	AB250212	—	DQ328726	56.7/100.0	Takahashi, unpublished. [20]	Sphaerulariidae
*Sphaerularia vespae *	AB300595	AB300595	AB300596	54.7/100.0	[77]	
Unidentified nematode 801L-022	EU880129	—	—	12.1/—	[74]	

**Anguinata**						
*Anguina tritici *	AY593913	JF826515	HO058555 DQ328723	57.6/92.9	Holterman et al., unpublished. Rao and Rao, unpublished. Rao et al., unpublished. [20]	Anguinidae
*Ditylenchus adasi *	EU669909	—	—	34.6/—	[19]	
*Ditylenchus angustus *	AJ966483	—	—	34.6/—	[16]	
*Ditylenchus destructor *		JX162205		50.0/99.5	[78]	
*Ditylenchus dipsaci *	AY593911	AY593911	JF327759	60.9/100.0	[76] Zhao 2011, unpublished.	
clone NTS_28S_061A_2_b4			KC558346		[79]	
*Ditylenchus drepanocercus *	JQ429768	JQ429774	JQ429772	48.7/89.3	[80]	
*Ditylenchus halictus *	AY589297			52.8/97.3	[68]	
*Ficotylus congestae *	EU018049			45.6/97.5	[81]	
*Halenchus fucicola *	EU669912	—	—	34.6/—	[19]	
*Pseudhalenchus minutus *	AY284638			34.6/—	[19]	
Unidentified entomoparasitic nematode SAS-2006 “*Neotylenchus*” sp.	—	—	DQ328725	—/85.6	[20]	

**“Tylenchina”: Tylenchidae**						
*Aglenchus agricola *	FJ969113	—	—	46.0/—	van Megen et al., unpublished.	Tylenchidae
*Aglenchus* sp.	—	—	JQ004996		[82]	
*Coslenchus costatus *	AY284581	—	—	45.5/—	[18]	
*Coslenchus* sp.	—	—	JQ005007		[82]	
*Filenchus annulatus *	JQ814880	—	JQ005017	46.4/—	[82]	
*Tylenchus davainei *	AY284588	—	—	33.9/—	[18]	

**“Tylenchina”: Tylodoridae**						
*Eutylenchus excretorius *	EU915487	EU915500	EU915490	35.8/—	[83]	Atylenchidae
*Cephalenchus hexalineatus *	AY284594	—	—	44.1/—	[18]	Tylodoridae

**“Tylenchina”: Boleodoridae**						
*Basiria gracilis *	EU130839	—	DQ328717	44.6/—	[84] [20]	Tylenchidae
*Basiria* sp. 3 TJP-2012	—	—	JQ004998	12.0/—	[82]	
*Boleodorus thylactus *	AY993976	—	—	46.7/—	[16]	
*Boleodorus* sp.	—	—	JQ005001		[18]	
*Neopsilenchus magnidens *	AY284585	—	—	45.6/—	[18]	
*Neopsilenchus* sp. 3 TJP-2012	—	—	JQ005020		[82]	
*Neopsilenchus* sp. 1 TJP-2012	—	—	JQ005018	11.9/—	[82]	

**“Hoplolaimina”: Merliniidae**						
*Nagelus leptus *	—	—	DQ328715	45.2/—	[20]	Telotylenchidae
*Nagelus obscurus *	EU306350	—	—		[17]	
*Pratylenchoides ritteri *	AJ966497	—	JX261964	48.7/—	[16] [85]	Pratylenchidae
*Psilenchus* cf. *hilarulus *	AY284593	—	EU915489	44.1/—	[18] [83]	Psilenchidae
*Scutylenchus quadrifer *	AY284599	—	—	41.5/—	[18]	Telotylenchidae
*Scutylenchus* sp.	—	JQ069956	—		[86]	

**“Tylenchina”: Ecphyadophoridae**						
*Ecphyadophora* sp. JH-2004	AY593917	—	—	33.7/—	[76]	Ecphyadophoridae
*“Ditylenchus” brevicauda *	AY284635	—	—	33.9/—	[18]	Anguinidae
*Malenchus andrassyi *	AY284587	—	—	32.3/—	[18]	Tylenchidae
*Ottolenchus discrepans *	AY284590	—	—	33.7/—	[18]	

**Criconematina**						
*Hemicriconemoides gaddi *	—	KC520471	KC520470	55.6/—	[87]	Criconematidae
*Hemicriconemoides pseudobrachyurus *	AY284622	—	—		[18]	
*Hemicycliophora lutosa *	—	GQ406237	GQ406240	53.2/—	[88]	Hemicycliophoridae
*Hemicycliophora thienemanni *	AY284628	—	—		[18]	
*Meloidoderita kirjanovae *	—	DQ768427	DQ768428	50.8/—	[89]	Sphaeronematidae
*Sphaeronema alni *	FJ969127	—	—		van Megen, unpublished.	
*Meloidoderita* sp.	GU253916	GU253917	JQ771954	50.8/—	[90] Cudejkova and Cermak, unpublished.	
*Tylenchulus semipenetrans *	AJ966511	FJ588909	FJ969710	57.5/—	[16] [91] [92]	Tylenchulidae

**“Hoplolaimina”: Belonolaimidae**						
*Belonolaimus longicaudatus *	AY633449	DQ672366	GQ896548	55.8/—	[68] [93] [94]	Belonolaimidae
*Ibipora lolii *	JQ771535	—	—	30.9/—	[95]	

**“Hoplolaimina”: Hoplolaimidae**						
*Carphodorus* sp.	JQ771538	—	JQ771550	41.3/—	[95]	
*Globodera pallida *	EU855119	EU85511	BM415342 BM415248 CV577211 CV577977 CV579301E U85511	93.6/—	Nowaczyk et al., unpublished. Opperman, unpublished [96].	Heteroderidae
*Heterodera glycines *	AF216579 BI704127 BI748392 CA940548 CB379240 CB379263 CB379850 CB380242 CB825296 CB825409 CB825970 CB935610 CK348871 CK348904 CK349175 CK352112	AF216579	AF133304 AF216579 BI704144 BI704144 BI749520 CA940190 CA940212 CA940243 CA940406 CA940424 CA940429 CA940589 CB238697 CB279977 CB299455 CB373844 CB373981 CB379125 CB379140 CB379219 CB379312 CB379439 CB379505 CB379696 CB379707 CB379996 CB380091 CB380241 CB824788 CB824878 CB825995 CB934877 CB934931 CB934950 CB934954 CK348525 CO036619 HM560850 JN684906	98.3/—	[97] [96]. [98] Yan and Davis, unpublished. [99] Ye et al., unpublished. Wei et al., unpublished.	
*Morulaimus* sp.	JQ771540	—	—	31.5/—	[95]	Belonolaimidae
*Radopholus similis *	AJ966502 AY912509 EF384224 EY190988 EY191076 EY191697 EY191883 EY192786 EY192788 EY193123 EY193253 EY194340 EY194464 EY194646 EY195472 FJ040398	AY912509 EF384224	EU555409 EY189839 EY190550 EY190620 EY190961 EY191066 EY191073 EY191135 EY191160 EY191173 EY191237 EY192021 EY192028 EY192080 EY192091 EY192247 EY192381 EY192472 EY192501 EY192526 EY192892 EY192907 EY193005 EY193037 EY193249 EY193314 EY193798 EY193897 EY193971 EY194395 EY194454 EY194530 EY195146 EY195204	97.5/—	[16] [100] Long et al., unpublished. [101] Holterman et al., unpublished. [102] [100] Zhao unpublished. [86]	Pratylenchidae
			EY195406			
			EY195408 EY195580 EY195889 EY195943 GQ281471 JN091962 JQ782249			
*Rotylenchulus reniformis *	JX406356	FJ374686	HM131884 FJ906072	59.4/—	[103] Rahman et al., unpublished. [104]	Rotylenchulidae

**“Hoplolaimina”: Pratylenchidae**						
*Dolichodorus* sp. WY-2006	DQ912918	—	—	33.9/—	[105]	Dolichodoridae
*Hirschmanniella loofi *	EU306353	EU620472	EU620469	51.6/—	[17] [106]	Pratylenchidae
*Macrotrophurus arbusticola *	AY284595	—		33.9/—	[18]	Telotylenchidae
*Meloidogyne arenaria *	U42342	U42342	U42342 AF023855 AF023856	99.2/—	Georgi and Abbott, unpublished.	Meloidogynidae
*Meloidogyne artiellia *	AF248477	AF248477	AF248477	99.2/—	[107]	
*Nacobbus aberrans *	AJ966494	DQ017473	U47557	49.0/—	[16] [108] [109]	Pratylenchidae
*Pratylenchus vulnus *	EU669955	JQ966892	BQ580554 CV198923 CV198995 CV199233 CV199349 CV199490 CV200136 CV200423 CV200464 CV200467 CV200471 CV200530 CV200687 CV200896 CV201004 CV201135 EL887566 EL887705 EL888035 EL888060 EL888174 EL888269 EL888739 EL888778 EL889241 EL889472 EL889797 EL889934 EL889934 EL889977 EL889994 EL890380 EL890701 JQ003993 JQ003994 JX047008	100.0/—	[19] [110] [96] [96] [111] Zhao, unpublished. [112]	
*Tylenchorhynchus dubius *	EU306352	—	DQ328707	53.2/—	[17] [20]	Telotylenchidae
*Tylenchorhynchus zeae *	—	EF519711	—		[113]	

*Clades of the tree, marked by boldface.

**Table 4:** Comparison of morphometrics in parasitic females of *Rubzovinema* sp. and *Rubzovinema  ceratophylla*.

Character	*Rubzovinema *sp. (this study)	*Rubzovinema ceratophylla* [26]
N	29	27
L	1278,6 (840–1570)	1265,1 (810–1840)
D	120,8 (85–145)	137,3 (62–200)
A	11,19 (7,9–16,1)	9,51 (6,4–16,8)
C	65,4 (31,4–100)	44,10 (10–86,4)
V%	96,4 (93,1–97,9)	95,44 (92–98,9)
Total length of stylet (St)	18,5 (14–22)	19,5 (18–21)
Length of distal edge of stylet	7,2 (5–8,7)	—
Distance between anterior end and excretory pore (Ex)	20,7 (10–31)	—
Distance between anterior end and nerve ring	61,2 (50–74,5)	
Total length of tail (Cd)	21,9 (10–42)	26,35 (14–47,5)
Distance between vulva and tail end	46,1 (23–75)	—
Distance between vulva and anus (V–A)	26,9 (13–40)	—

All measurements are in *μ*m and in the form mean (range).

**Table 5:** Comparison of morphometrics of parasitic females in *Spilotylenchus* sp. and *Spilotylenchus maisonabei*.

Characters	*Spilotylenchus *sp. (this study)	*Spilotylenchus maisonabei* [23]
N	2	6
L	1,600–1,840	1,244 (1,200–1,320)
D	155–160	125 (107–160)
A	10.3–11.5	10.3 (7.5–12)
C	167.3–177.8	84.4 (64.5–121)
V%	97.4–97.7	96.2 (95.8–96.5)
Total length of stylet (St)	9.5–9.8	9-10
Distance between anterior end and excretory pore	1.5–15.5	23.3 (20–28)
Distance between anterior end and nerve ring	—	52–54
Total length of tail (Cd)	9–11	15.4 (10–19)
Distance between vulva and tail end	41.5–43	47 (42–52)
Distance between vulva and anus (V–A)	32-33	—

All measurements are in *μ*m and in the form mean (range).

**Table 6:** Comparison of morphometrics of parasitic females in *Psyllotylenchus* sp. and *Psyllotylenchus viviparous*.

Character	*Psyllotylenchus* sp. (this study)		*Psyllotylenchus viviparous* [25]	
	Gamogenetic	Parthenogenetic	Gamogenetic	Parthenogenetic
N	3	7	8	10
L	1,016.7 (900–1,100)	446 (420–500)	1,000 (840–1,480)	500 (360–840)
D	81.3 (79–84)	70 (60–80)	77 (62–115)	60 (54–100)
A	12.5 (11.1–13.3)	6.25 (5.6–7)	—	—
C	64.3 (60–68.2)	40.15 (37.1–43.5)	—	—
V%	95.1 (95–95.4)	93.3 (90–95.3)	—	—
Total length of stylet (St)	17.5 (17–18,5)	5.25 (4–6)	17 (15–20)	7 (5–8)
Length of the distal edge of stylet	8.6 (8-9)	—	—	—
Distance between anterior end and excretory pore	26.5 (25–31.5)	17.5 (15–19.5)	23 (13–33)	22 (14–46)
Distance between anterior end and nerve ring	—	51.7 (50–55)	—	—
Total length of tail (Cd)	15.8 (15–17)	11.1 (10.5–11.5)	25 (17–35)	9 (1–17)
Distance between vulva and tail end	48 (45–51)	30.5 (19.7–55)	56 (37–71)	52 (40–104)
Distance between vulva and anus (V–A)	30.8 (29–31.5)	13.5 (11.7–21.6)	—	—

All measurements are in *μ*m and in the form mean (range).

**Table 7:** Results of tree topology tests for alternative hypotheses on (1) the initial divergence of Tylenchida (Figure 4) and on (2) the relationships within the monophyletic branch that includes the studied group of nematodes parasitizing fleas (designated by asterisk).

Topology	Rank	obs	au	np	bp	pp	kh	sh	c-ELW
1									
(((H,An),T),o)	1	−1.8	0.787	0.415	0.402	0.804	0.663	0.969	0.4197
((An,(H,T)),o)	2	4.1	0.326	0.198	0.205	0.013	0.254	0.623	0.1848
((H,(An,T)),o)	3	6.9	0.061	0.013	0.014	0.001	0.101	0.492	0.0186

2									
((((∗,Al),P),Ds),o)	1	−1.8	0.787	0.415	0.402	0.804	0.663	0.969	0.4197
((((∗,P),Al),Ds),o)	2	1.8	0.495	0.242	0.247	0.130	0.337	0.813	0.2249
(((∗,(Al,P)),Ds),o)	3	2.7	0.371	0.110	0.105	0.052	0.243	0.824	0.1209
((∗,((Al,P),Ds)),o)	6	15.7	0.063	0.024	0.025	1*e* − 007	0.053	0.153	0.0272
(((∗,Ds),(Al,P)),o)	7	18.3	0.013	0.002	0.002	9*e* − 009	0.020	0.096	0.0028

Al: Allantonematidae, An: Anguinata, Ds: *Deladenus siricidicola*—*D. proximus* group, H: Hexatylina, P: Parasitylenchidae, T: Tylenchina, o: outgroup.
